# Impact of tissue heterogeneity on intracranial Cesium‐131 brachytherapy dosimetry: A patient‐specific Monte Carlo Study

**DOI:** 10.1002/acm2.70872

**Published:** 2026-10-29

**Authors:** Tirthraj Adhikari, Jae W. Jung, Sherif M. Gadoue, Puja Sharma, Tomas Montegegro, Farhad Jafari, Clara Ferreira

**Affiliations:** ^1^ University of Minnesota Minneapolis Minnesota USA; ^2^ East Carolina University Greenville North Carolina USA; ^3^ University of Texas MD Anderson Cancer Center Houston Texas USA; ^4^ Kathmandu University Nepal; ^5^ Loyola University Medical Center Maywood Illinois USA

**Keywords:** Cesium‐131, GammaTile brachytherapy, MC simulation, tissue heterogeneity

## Abstract

**Background:**

AAPM TG‐43‐based dose calculations for intracranial cesium‐131 (^131^Cs) brachytherapy ignore patient‐specific heterogeneities, which can affect dose accuracy. This study investigates the dosimetric impact of these heterogeneity effects using Monte Carlo (MC) simulations and patient‐specific imaging data.

**Purpose:**

To quantify the impact of tissue heterogeneity on dose distributions in intracranial brachytherapy using ^131^Cs sources and to evaluate the accuracy of AAPM TG‐43‐based treatment planning system (TPS) calculations.

**Methods:**

TG‐43 dosimetric parameters for the ^131^Cs source were independently characterized and benchmarked against published consensus data. MC simulations using MCNP were performed in both homogeneous water phantoms and heterogeneous voxelized lattice phantoms derived from CT DICOM RT data. Single and multiple‐seed configurations were investigated. TPS and MC dose distributions were compared using dose‐volume histogram (DVH) metrics such as (V_60_, V_100_, D_2cc,_ and mean dose), isodose distributions, dose difference maps, and local and global dose difference analyzes. Spatial agreement was quantified using gamma analysis.

**Results:**

Strong agreement was observed between TPS and MC calculations in homogeneous media. However, heterogeneous patient‐specific MC simulations revealed systematic differences in DVH metrics for the target volume and organs at risk, including the skull and skin. Local dose differences revealed pronounced deviations near high‐density bony regions, where dose enhancement effects were observed, while global dose difference analyzes demonstrated an overall dosimetric bias. Gamma pass rates decreased in heterogeneous regions, consistent with these spatial dose discrepancies. Compared with the MC simulations, the TG‐43‐based TPS overestimated the mean dose to the target by approximately 23.63±4.97%, V_60_ and V_100_ by approximately 24.42±4.97%, and 23.75±4.97%, respectively, and the mean dose to the skin by approximately 73.60±4.97%, while underestimating the mean dose to the skull by 220.90±4.97%.

**Conclusion:**

Although the TG‐43 formalism provides accurate dose estimates in homogeneous media, it introduces clinically meaningful local and global dosimetric deviations in heterogeneous intracranial environments. Careful consideration is required when reporting doses to the target and OARs, particularly given the potential for substantially higher doses to the irradiated skull in certain cases (up to 3.2‐fold in this study), as well as the possibility of target underdosage within the high‐risk clinical target volume (HR‐CTV). Patient‐specific MC modeling highlights the importance of incorporating tissue heterogeneity into intracranial brachytherapy dose calculations to improve dosimetric accuracy and optimize treatment planning.

## 1 INTRODUCTION

Tile‐based intracranial brachytherapy has emerged as an effective treatment modality for primary and recurrent brain tumors, enabling highly localized dose delivery while minimizing radiation exposure to surrounding healthy tissues.^1^, ^2^, ^3^ Among available radioisotopes, Cesium‐131 (^131^Cs) has attracted increasing clinical interest because of its short half‐life and favorable dosimetric characteristics, which facilitate rapid dose deposition and may reduce treatment burden to patients.^4^, ^5^, ^6^

Accurate dose calculation is fundamental to the success of brachytherapy treatments. In current clinical practice, dose calculations are predominantly based on the American Association of Physicists in Medicine (AAPM) Task Group 43 (TG‐43) formalism.^7^ Although TG‐43 remains computationally efficient and widely adopted, it assumes a homogeneous water medium and neglects patient‐specific heterogeneities, including variations in tissue composition and density. Consequently, the formalism does not account for clinically relevant factors, including bone, air cavities, soft‐tissue interfaces, seed shielding, and interseed attenuation, all of which can influence dose distributions in intracranial brachytherapy.^8^

The importance of accurate post‐implant dosimetry is particularly evident in tile‐based intracranial brachytherapy, in which 60 Gy is typically prescribed such that 90% of the prescribed dose encompasses HR‐CTV. Accurate post‐implant dosimetry is essential for verifying adequate HR‐CTV coverage, identifying hot and cold spots, protecting organs at risk (OAR), detecting potential source migration due to target shrinkage, and ensuring overall treatment quality and patient safety. Because low‐energy brachytherapy sources produce steep dose gradients, even relatively small inaccuracies in dose calculation may lead to clinically meaningful deviations in delivered dose. Therefore, the development and validation of accurate dosimetric methodologies are critical for reliable treatment evaluation and optimal clinical outcomes.

The effect of tissue heterogeneity in low‐energy (LE, < 50 keV) brachytherapy has received considerable attention since the publication of AAPM TG‐186 in 2012,^8^ which advocated the use of model‐based dose calculation algorithms (MBDCAs) that explicitly account for tissue composition and density. The report highlighted the pronounced differences in mass energy absorption coefficients among high atomic number materials at low photon energies, where photoelectric interactions dominate. In contrast, these differences are substantially reduced at higher photon energies (> 50 keV). Consequently, dose distributions from low‐energy brachytherapy sources are particularly sensitive to tissue heterogeneities that may significantly alter photon transport and dose deposition.

Subsequent investigations have confirmed the dosimetric impact of tissue heterogeneity in brachytherapy. Previous MC and model‐based dose calculation studies have reported dose differences ranging from approximately 3% to 25% between dose‐to‐medium and dose‐to‐water calculations, while TG‐43‐based methods have shown deviations of up to 82% when compared with heterogeneous medium‐based calculations and MC simulations^9^, ^10^, ^11^, ^12^, ^13^, ^14^, ^15^ Yang and Rivard demonstrated the influence of tissue heterogeneity by modeling photon sources with energies between 20 and 400 keV and found that TG‐43 overestimated target dose while underestimating dose to bone and normal tissues in lung treatments.^16^ Similarly, Sutherland et al. reported a 36% difference in D_90_ when patient‐specific tissue compositions were incorporated into ^125^I lung brachytherapy calculations.^17^

Although substantial evidence exists regarding heterogeneity effects for ^103^Pd and ^125^I implants, far fewer studies have investigated these effects for ^131^Cs brachytherapy. Nevertheless, previous studies have demonstrated that tissue heterogeneity can significantly influence photon transport for low‐energy sources such as ^131^Cs, where photoelectric interactions dominate.^18^, ^19^, ^20^ These effects become particularly pronounced near high‐density structures such as the skull and at tissue‐air interfaces, where attenuation and scattering processes substantially modify local dose deposition. These observations emphasize the importance of patient‐specific dosimetric modeling for intracranial brachytherapy.

MC techniques are widely regarded as the gold standard for radiation transport calculations because they accurately model particle interactions within complex geometries and heterogeneous media without introducing the simplifying assumptions required by analytical transport models.^21^, ^22^, ^23^ Unlike conventional water‐based dose calculations, MC simulations explicitly account for variations in elemental composition and mass density, enabling realistic and patient‐specific dose estimations. Although MC methods have been extensively employed to investigate heterogeneity effects in brachytherapy and external beam radiation therapy applications, many published studies have relied on simplified geometries, lacked comprehensive validation, or did not include experimental verification. To our knowledge, no previous study has incorporated patient‐specific tissue heterogeneity into a fully validated MC framework for the dosimetric evaluation of intracranial ^131^Cs seed implants. This gap highlights the need for a comprehensive assessment of heterogeneity effects in brain brachytherapy using patient‐specific MC modeling.

Complementing these developments, recent research has focused on establishing standardized commissioning and validation datasets for MBDCAs in accordance with TG‐186 recommendations and the AAPM WGDCAB Report 372. Mansour et al.,^24^ provided reference datasets for commissioning MBDCAs in electronic brachytherapy, while Akbari et al.^25^ developed three‐dimensional reference dosimetric datasets to facilitate the implementation of MBDCAs in permanent prostate implant brachytherapy. However, analogous reference datasets and standardized validation cases are not currently available for low‐energy intracranial brachytherapy applications, including GammaTile™, representing an important unmet need.

Motivated by these gaps, we developed a series of progressively more realistic test cases for commissioning and validating model‐based dose calculation approaches for intracranial brachytherapy. The proposed test cases span increasing levels of complexity, ranging from a single ^131^Cs source in a homogeneous water medium to multiple‐source configurations within patient‐specific anatomical geometries. This framework provides a systematic pathway for evaluating the dosimetric effects of tissue heterogeneity while facilitating benchmarking of advanced dose‐calculation methodologies. Using an in‐house computational workflow, we systematically quantified the dosimetric impact of tissue heterogeneity in tile‐based intracranial brachytherapy.

The primary objective of this study was to develop and validate a comprehensive dosimetric framework that integrates TG‐43 benchmarking, MC simulation, and patient‐specific heterogeneous modeling. Using this framework, we quantified dosimetric differences between TG‐43‐based TPS calculations and material‐specific MC simulations in clinically realistic intracranial environments. In addition, we established a hierarchy of validation cases that can serve as reference benchmarks for future commissioning and verification of model‐based dose calculation algorithms. The spatial and clinical significance of these differences was evaluated using DVH metrics, gamma‐index analysis, dose‐difference mapping, and profile‐based comparisons. Collectively, the results reinforce the importance of accounting for tissue heterogeneity in low‐energy brachytherapy, where strong photoelectric absorption and atomic number dependence make dose distributions particularly sensitive to variations in material composition and density.

## 2 METHODS

### 2.1 MC benchmarking and experimental validation

#### 2.1.1 MC benchmarking

In this study, we performed MC simulations using MC N‐particle (MCNP) [RSICC‐Los Alamos National Laboratory, USA] version 6.3 to model photon transport and calculate absorbed dose distributions for intracranial brachytherapy using ^131^Cs sources employed in the collagen Tile brain implant. MCNP is a general‐purpose MC radiation transport code widely used in medical physics because of its accurate modeling of coupled particle transport and detailed treatment of low‐energy photon interactions.^26^ The simulation methodology followed the recommendations of the AAPM TG‐43 report for brachytherapy source dosimetry,^7^ and the corresponding checklist is provided in Table 6.

To benchmark the MCNP model, the TG‐43 dosimetric parameters: Air‐Kerma Strength (AKS) ($cGy.cm^{2}.h^{-1}or\;U),$ dose rate constant Λ (cGyh^−1^ U^−1^), radial dose function (g(r)), and anisotropy function (F(r,θ)) for a ^131^Cs (IsoRay Model CS‐1, Rev2) source were first characterized from simulation data and compared with published TG‐43 consensus data. The ^131^Cs source with strength 3.5 U was modeled as specified in AAPM TG‐43U1S2, as displayed in Figure 1.

**FIGURE 1:**
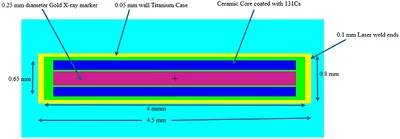
MCNP‐generated ^131^Cs Source model employed in this work.

The Isoray™ ^1^ ^3^ ^1^Cs brachytherapy seed (model Cs‐1 Rev2) [GT Medical Technologies, Inc., the USA] has a total physical length of 4.5 mm, with 0.1 mm thick end caps and an outer diameter of 0.8 mm (Figure 2). The seed's active core is encapsulated within a 0.05 mm thick titanium (Ti) ($\rho =4.54\;g/cm^{3})$ wall and contains a 0.25 mm diameter gold (Au) ($\rho =19.3\;g/cm^{3})$ X‐ray marker at its center. This marker is coated with glass and ceramic ($\rho =2.4\;g/cm^{3})$ materials, and the inner core has an outer diameter of 0.65 mm. The active length of the source is 4 mm. The coatings on the active core may add slight thickness to both ends of the X‐ray marker, making 4.1 mm a reasonable and representative value for dosimetric modeling. The void within the Ti capsule is filled with argon ($\rho =1.784\;x\;10^{-3}g/cm^{3})$. The National Nuclear Data Center (NNDC)^27^ ^131^Cs source photon spectrum 4.11 keV (8.6%), 29.46 keV (21.1%), 29.78 keV (38.9%), 33.56 keV (3.63%), 33.62 keV (02%, and 34.42 keV (2.13%) was used for simulation.

**FIGURE 2:**
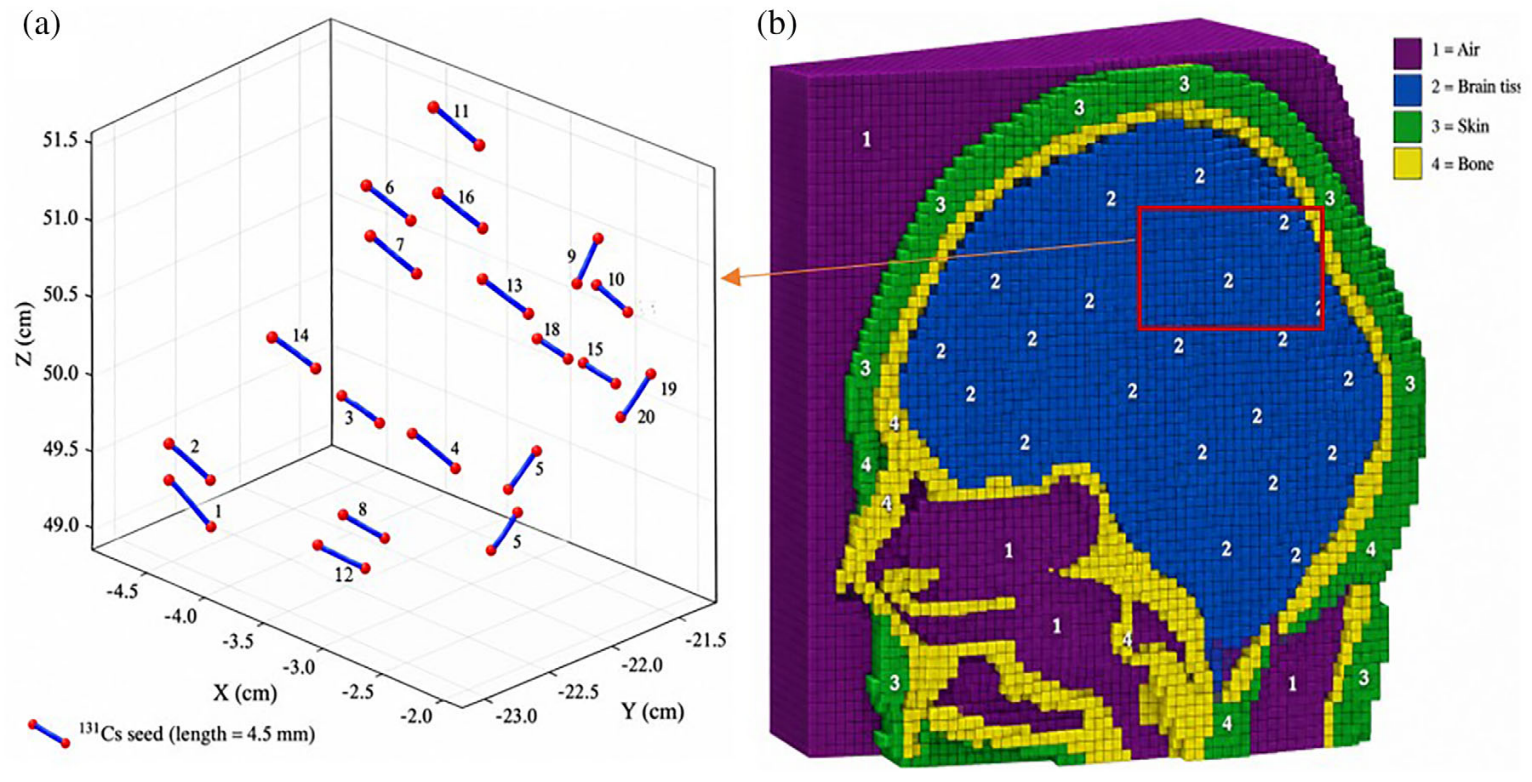
a. Representative three‐dimensional distribution of ^131^Cs seed positions in the phantom used in this work. Each point represents the (x, y, z) location of a seed used in the test case. b. Representative 3D voxelized lattice phantom geometry created in MCNP. The numbers represent the assigned material universes: 1 = air, 2 = brain tissues, 3 = skin, and 4 = bone. Each box represents 0.043x 0.043 x 0.100 cm^3^ voxel.

The elemental composition and density of the materials of ^131^Cs source used for simulation in this work is presented in Table 1. The MCNP6.3's default cross‐section library, EPICL2017, was used. The geometry of the source seed was verified in MC's geometry plotter as presented in Figure 1.

**TABLE 1:** Elemental composition and density of the materials of ^131^Cs source used in this work.

Material	Density [g/cm^3^]	Element	Fraction [‐]	Reference
Gold	19.23	Au	1	NIST
Ceramic	2.4	B O Na Al Si K	−0.040064 −0.539561 −0.028191 −0.011644 −0.377219 −0.003321	PNNL
Argon	1.784 × 10^−3^	Ar	1	NIST
Titanium	4.54	Ti	1	NIST
Polystyrene	1.06	H C	−0.077426 −0.922574	PNNL
Glass	2.2	O Si	−0.532570 −0.467430	PNNL
Collagen	1.003	H C N O	0.311475 0.497268 0.103825 0.087432	NCBI
PDMS	0.965	C O Si	−48.5 −28.5 −23.4	NCBI
Water	1	H O	0.333 0.667	NCBI
Air	1.205 × 10^−3^	C N O Ar	−0.000124 −0.755268 −0.231781 −0.012827	TG‐43

AKS was simulated using a geometry consistent with the National Institute of Standards and Technology (NIST) Wide‐Angle Free‐Air Chamber (WAFAC) reference conditions with relative humidity of 40%. In this approach, the air kerma per simulated history was scored in a small dry air‐filled tally cell located 10 cm from the source. The MCNP *F4* tally was used to calculate the average track‐length fluence (particles/cm^2^), which was then multiplied by the mass energy‐transfer coefficient of air $(\mu_{tr(E)/\rho }$), to obtain the air kerma rate per unit source strength (U·h per particle) by running 2 × 10^9^ history. The relative statistical uncertainty for the AKS simulation was 0.03%(k = 1).

The dose rate constant was simulated in a spherical water phantom with a 15 cm radius, following the AAPM TG‐43 formalism. Tallies were scored at radial distances ranging from 0.5 cm to 7.0 cm. Concentric ring tally cells measuring 0.1 cm × 0.1 cm × 0.1 cm were positioned at the required radial distances and polar angles relative to the source as shown in supplementary material Figure S1. The dose rate constant (Λ) was determined as the ratio of the dose rate at the reference position, $P(r=1\;cm,\;\theta =90^{0}\;)$, to the AKS (S_K_). We used the MCNP F6 (energy deposition) tally to score the absorbed dose by calculating the average energy deposited in each tally cell (MeV/g‐histories). The number of particle histories simulated was 2 × 10^9^. The MC relative statistical uncertainty was 0.02%(k = 1) in this simulation.

To simulate the radial dose function, which accounts for dose fall‐off in the transverse plane due to photon scattering and attenuation, the *F6* tally was scored in the same geometry as the dose rate constant by placing 1mm^3^ water detector in a 15 cm radius water phantom from 0.25 to 7 cm with 10‐degree increments from 0^0^ to 180^0^ as shown in Supplementary Material Figure S1. A similar procedure was adopted to simulate the anisotropy function, which describes the variation in dose as a function of polar angle relative to the transverse plane, using the same simulation as that run for the radial dose function at angles varying from 0^0^ to 90^0^ and distance from 0.5 to 7 cm. The number of histories simulated was 2 × 10^9,^ and the maximum type A uncertainty in the MC calculation was 0.02% and 0.05%, respectively, for the radial and anisotropy functions.

Following the simulation of AKS, dose rate constant, radial dose function, and anisotropy function, the dose rate was estimated by employing the TG‐43 formalism.^7^

#### 2.1.2 MC experimental validation

To validate the MCNP code, in‐house calibrated EBT4 Gafchromic films [Ashland, NJ, USA] were irradiated at clinically relevant depths of 0.8 cm and 1.3 cm from the ^131^Cs seed axis. The 0.8 cm measurement corresponds to the prescription depth (0.5 cm) beyond a 0.3 cm collagen tile, while the 1.3 cm measurement represented the skin depth in intracranial implant scenarios. For the 0.8 cm measurement, the film was positioned between two bone‐equivalent phantoms ($\rho =1.85\;g/cm^{3})$ and for the 1.3 cm measurement, it was positioned between a bone‐equivalent phantom and a Solid‐Water (SW) phantom ($\rho =1.01\;g/cm^{3})$ as shown in supplementary material Figure S2a. The overall film measurement Type B uncertainty was 3%. This uncertainty resulted from factors such as small gaps between phantom layers, EBT4 film calibration uncertainty, uncertainty in source strength, seed positioning, film handling, development, scanning, and differences in material properties and was assessed as per AAPM TG‐138.^28^

We modeled the identical experimental geometry in MCNP and performed dose calculations by running 2 × 10^9^ particle histories, as shown in Figure supplementary material S2c. The Type A relative statistical uncertainty was 2.35%.

### 2.2 Patient‐specific brain model

An IRB‐approved and anonymized Digital Imaging and Communications in Medicine (DICOM) dataset was utilized to create a simplified, heterogeneous voxelized phantom using the Computational Environment for Radiological Research (CERR) and MATLAB R2024b (MathWorks, Natick, MA, USA). This virtual patient‐specific brain model was constructed using the post‐implant CT images of a real patient, ensuring the model's clinical relevance.

The CT datasets were imported into CERR, integrated with MATLAB, and converted into 3D datasets. Then the HU and corresponding density information of the tissues were extracted for the whole CT image, which was 512 × 512 × 177 voxels. Then, the threshold HU values for the tissues were assumed based on the corresponding densities, according to ICRU‐44, as presented in Table 2. Material universes were then assigned for the tissues of interest, namely, scalp, skull, brain tissue, and air, and the robustness of the assignment was verified slice by slice by comparing and visualizing in axial, coronal, and sagittal views with the original CT image, as shown in Figure S3 (axial view). Then the MCNP input deck was created, and the patient‐specific brain model was also verified using MCNP's geometry plotter, as shown in Figure 2b. The HU‐to‐Material mapping uncertainty was 3.50%.

**TABLE 2:** The threshold Hounsfield Units employed for voxelized phantom construction.

Material	Hounsfield number (HU)	Material universe	Density gm/cm^3^
Air	< ‐ 900	1	1.21 × 10^−3^
Brain tissue	>‐900 < 60	2	1.04
Skin	>61 < 120	3	1.09
Bone	>120	4	1.92

Due to the memory limitations of MCNP, it was not possible to simulate the whole brain; therefore, a sub‐volume of dimensions 22.06 × 22.06 × 17.62 cm^3^ was used by truncating the original CT data to 149 × 135 × 67 voxels in the X, Y, and Z directions, respectively. The dose scoring grid was kept at the same resolution as the original patient imaging data, with voxel sizes of 0.04 cm × 0.04 cm × 0.10 cm. The voxelization/volume averaging uncertainty was 2% which depends on the distance from the seed.

In accordance with the recommendations of TG‐186, which acknowledges the challenge of assigning voxel‐by‐voxel material composition from CT and allows the use of structure‐based uniform densities for commissioning and benchmarking, a uniform density was assigned to each anatomical structure in this reference dataset.

This simplification facilitated debugging, minimized potential sources of error, and enabled clearer identification of computational discrepancies by avoiding additional complexities associated with continuously varying material densities. Although this approximation may not fully reflect the true heterogeneity of tissues and high‐density materials, it provides a reproducible and controlled basis for cross‐algorithm evaluation, consistent with practices used in other published test cases.^24^, ^25^

### 2.3 Clinical test case

This study included four benchmark test cases, summarized in Table 3, all based on the ^131^Cs sources used in collagen tile‐based surgically targeted radiation therapy implants for intracranial brachytherapy. The suite of test cases spans a range of geometrical and physical complexities, from simplified reference configurations—such as a single ^131^Cs source in a homogeneous water medium to clinically representative scenarios involving multiple GammaTile implants placed along a postoperative resection cavity within a heterogeneous brain phantom.

**TABLE 3:** Overview of the test cases studied.

Test Case number	Description	Seed configuration	Phantom
1	Single seed in water (TG‐43)	Single seed	Water
2	Single seed in phantom	Single seed	CT‐based voxelized phantom
3	Multi‐seed (TG‐43)	20 seeds	Water
4	Multi‐seed virtual patient	20 seeds	CT‐based full tissue phantom without bioabsorbable materials

The first case consisted of a single ^131^Cs seed in a homogeneous water phantom, in which all voxels were assigned water ($=1\;g/cm^{3})$ to reproduce the homogeneous conditions assumed by the TG‐43 formalism. This case provided a baseline for evaluating the source model under uniform conditions. The second case consisted of a single seed positioned within the CT‐derived, fully heterogeneous patient phantom. This configuration was used to evaluate the effect of patient‐specific tissue heterogeneity on the dose distribution from an individual seed. The third case included 20 seed positions in a homogeneous water phantom under TG‐43 reference conditions. To isolate the contribution of each seed and maintain consistency with the TG‐43 formalism, the seeds were activated individually, with the remaining seeds treated as inactive. Thus, the calculated dose represented the superposition of individual seed contributions without interseed attenuation effects. The fourth case included all 20 seeds positioned according to the post‐implant CT geometry within the CT‐derived heterogeneous patient phantom. This configuration represented the clinically relevant treatment scenario and was used to quantify the effects of tissue heterogeneity and high‐Z materials on the dose distribution and treatment‐planning metrics. A minimum of (1 × 10^9^) particle histories were simulated for each case using benchmarked and validated MCNP version 6.3.

The same well‐characterized ^131^Cs brachytherapy source used in the GammaTile implant was modeled in all four cases to ensure consistency across simulations following the approach described by Rivard et al.^29^ The seed coordinates used in patient‐specific simulations were derived from the post‐implant CT images and are listed in Table 4, and a representative voxel phantom with a 3D distribution of seeds is displayed in Figure 2. Source positioning (seed inversion) related uncertainties were 1.5%.

**TABLE 4:** Positions of 20 seeds in the brain for the clinical case extracted from post‐implant CT.

Source number	X (cm)	Y(cm)	Z (cm)	Source number	X (cm)	Y(cm)	Z (cm)
1	−2.88	−22.76	50.19	11	−3.16	−21.76	51.5
	−2.48	−22.76	50.19		−3.53	−21.76	51.65
2	−3.02	−22.76	49.47	12	−2.21	−21.76	50.52
	−3.42	−22.76	49.47		−2.07	−21.76	50.89
3	−3.48	−22.76	50.34	13	−4.19	−21.76	49.57
	−3.88	−22.76	50.39		−4.58	−21.76	49.64
4	−4.41	−22.76	48.97	14	−3.21	−21.76	49.31
	−4.75	−22.76	49.18		−3.58	−21.76	49.45
5	−3.99	−23.15	49.56	15	−2.09	−22.5	50.23
	−4.35	−23.15	49.73		−2.34	−22.5	49.92
6	−3.56	−22.33	48.9	16	−3.83	−21.42	50.91
	−3.96	−22.33	48.9		−3.45	−21.42	50.78
7	−2.17	−22.33	50.97	17	−3.98	−21.42	50.32
	−2.57	−22.33	51.04		−4.37	−21.42	50.44
8	−3.22	−22.24	51.15	18	−2.97	−21.25	50.13
	−3.61	−22.24	51.26		−3.35	−21.25	50.26
9	−2.64	−21.89	49.61	19	−2.48	−21.25	50.51
	−2.84	−21.89	49.26		−2.83	−21.25	50.7
10	−2.2	−21.76	51.26	20	−2.4	−21.25	50.16
	−2.6	−21.76	50.19		−2.67	−21.25	49.87

Collectively, the proposed test cases established a systematic framework for evaluating model‐based dose calculation algorithms in permanent intracranial brachytherapy. By progressively incorporating source interactions, implant geometry, and tissue heterogeneities characteristic of collagen Tile‐based brachytherapy, the framework enables rigorous benchmarking of dose calculation methods and supports the development and validation of advanced treatment planning approaches for ^131^Cs brain brachytherapy.

### 2.4 Patient‐specific mc simulation

The radiation sources were modeled according to the ^131^Cs brachytherapy seeds embedded within the bioresorbable collagen tiles used in GammaTile implants. Each tile contains multiple ^131^Cs sources arranged in a fixed geometric configuration within a collagen matrix carrier designed for placement along the surgical resection cavity, with a typical activity of 3.5 U per seed. The computational model included explicit geometric representations of the source encapsulation, internal active material, and surrounding collagen carrier to capture attenuation and scattering effects within the implant accurately.

Photon transport was simulated using the low‐energy photon physics models available in MCNP version 6.3. The simulations accounted for the primary photon interaction processes relevant to low‐energy brachytherapy sources, including photoelectric absorption, coherent (Rayleigh) scattering, incoherent (Compton) scattering, and characteristic X‐ray fluorescence emission. These interactions are particularly important for ^131^Cs photons, whose relatively low photon energies result in increased sensitivity to material composition and density variations due to the dominance of the photoelectric effect. The International Commission on Radiation Units and Measurements (ICRU) report 44 tissue elemental compositions were used and presented in Table 5 .

**TABLE 5:** Elemental composition and mass density of tissues used in the tissue assignment scheme.

Tissue	Elemental composition (percentage by mass)												Density (ρ) g/cm^−3^
	H	C	N	O	Ar	Na	P	S	Cl	K	Mg	Ca	
Air	–	1.50 10^−4^	0.78	0.21	4.76 10^−3^	–	–	–	–	–	–	–	1.2110^−3^
CSF	11			88		0.3			0.35		0.003	0.004	1.05
Gray Matter	10.7	14.5	2.2	71.2	–	0.2	0.4	0.2	0.3	0.3	–	–	1.040
White Matter	10.7	14.5	2.2	71.2	–	0.2	0.4	0.2	0.3	0.3	–	–	1.040
Brain	10.7	14.5	2.2	71.2	–	0.2	0.4	0.2	0.3	0.3	–	–	1.040
Blood	10.2	11.0	3.3	74.5	–	0.1	0.1	0.2	0.3	0.3	–	–	1.060
Scalp (Skin)	10.0	20.4	4.2	64.5	–	0.2	0.1	0.2	0.3	0.1	–	–	1.090
Skull (cortical bone)	3.4	15.5	4.2	43.5	–	0.1	10.3	0.3	–	–	0.2	22.5	1.920

**TABLE 6:** MC reporting checklist based on AAPM TG‐268^28^ for MCNP 6.3 simulation of a ^131^Cs BT source.

Checklist Item #	Item Name	Description	MCNP Implementation for ^131^Cs	References
2,3	Code, version/release date	Name and version of the MC code used, including release date.	MCNP version 6.3, released Sept 2022.	MCNP 6.3 Manual.
4,17	Validation	Reference to validation efforts, benchmark comparisons, and code testing.	Validated against AAPM TG‐43 dosimetry parameters for ^131^Cs.	AAPM TG‐43U1, TG‐268.
5	Timing	Computational resources, CPU time, parallelization info.	Simulations run on a 16‐core workstation, ∼8 hours per full run.	AAPM TG‐268.
8	Source description	Details of source model: geometry, energy spectrum, spatial distribution.	Modeled ^131^Cs seed with encapsulation; average photon energy: 30 keV.	IsoRay source specs, AAPM TG‐43U1S2.
9	Cross‐sections	Nuclear/atomic data libraries used	ENDF/B‐VIII; EPICS2017 photon cross‐section library used in MCNP 6.3.	MCNP Manual.
10	Transport parameters	Cutoff energies, particle types, and physics models used.	Photon‐only transport, 4.11 keV cutoff; electron transport disabled.	MCNP Manual.
11	VRT and/or AEIT	Variance Reduction Techniques (VRT), Auto‐importance/Energy Importance (AEIT).	Geometry splitting and Russian roulette are applied near the scoring region.	AAPM TG‐268, MCNP Manual.
12	Scored quantities	What was scored? (e.g., dose, energy fluence, particle flux).	Absorbed dose (MeV/g) scored using F6, F4, and mesh tally in water phantom.	AAPM TG‐268.
13,18	# histories/statistical uncertainty	Number of histories run and associated statistical uncertainties (± 1σ).	1×10^9^ histories; statistical uncertainty < 2% within 5 cm of source.	AAPM TG‐268.
14	Statistical methods	How were uncertainties calculated? What statistical estimators were used?	Relative error reported from MCNP tally output; Figure of Merit (FOM) used.	MCNP Manual, AAPM TG‐268.
15,16	Postprocessing	Any postprocessing applied to results (e.g., smoothing, conversion to dose).	Dose rate normalized to 1 U source strength; processed with Python scripts.	AAPM TG‐43U1, in‐house software.

A photon energy cutoff of 4.11 keV was applied to ensure accurate modeling of low‐energy secondary photons while maintaining computational efficiency, as this energy does not contribute to dose distribution as it is absorbed by the titanium capsule. Photon cross‐section data were obtained from the standard evaluated nuclear data libraries (ENDF/B‐VIII) distributed with MCNP 6.3.

The absorbed dose was estimated by calculating the collision kerma within the scoring medium. Photon fluence spectra were recorded within 3D voxelized regions using the MCNP's energy deposition (tmesh tally). The fluence values were then converted to collision kerma through multiplication by the corresponding mass energy‐absorption coefficients for the scoring material.

For 3D dose calculations, the computational phantom was discretized into a voxel grid surrounding the implant region. The voxel resolution was selected to balance spatial accuracy and computational efficiency, enabling high‐resolution dose mapping near the GammaTile sources where steep dose gradients were present.

Each simulation was performed with a sufficiently large number of particle histories to achieve acceptable statistical precision in the dose estimates. The number of simulated histories was selected such that the relative statistical uncertainty in regions of clinical interest remained below approximately 1%. Statistical uncertainties were evaluated using the standard history‐by‐history variance estimation in MCNP.

Absolute dose calculations were performed by normalizing simulation results to the AKS of the ^131^Cs sources. The AKS per particle history factor ($S_{k}^{hist})$ was determined using a geometry consistent with the National Institute of Standards and Technology wide‐angle free‐air chamber (WAFAC) reference configuration, as in the MC benchmarking process. In this approach, the air kerma per simulated history was scored in an air voxel positioned 10 cm from the source. The air‐kerma strength per history factor was calculated as:

$\;S_{k}^{hist}={\dot{K}}_{\delta }\;\left(d\right)\times d^{2}\times k_{r^{2}}$, where ${\dot{K}}_{\delta }\left(d\right)$ is the air kerma per history due to photons with energies greater than δ=4.11 keV in a 0.04 × 0.04 × 0.10 cm^3^ voxel at a distance d = 10 cm from the source, and the factor $k_{r^{2}}$ corrects for inverse‐square effects over the scoring voxels. The correction factor used in this study was 1.02.

The calculated $S_{k}^{hist}$ factor was then used to convert the MC results into absolute dose values expressed in units of gray (Gy), providing reference dosimetric data for benchmarking dose calculation algorithms in GammaTile intracranial brachytherapy.

### 2.5 Dose scoring and comparison metrics

DVH matrices were compared using the Computation Environment for Radiological Research (CERR) GUI between the TG‐43‐based TPS and MC. To quantify the voxel‐by‐voxel differences in dose distributions, two 3D dose comparison metrics were used as follows:

(1) $$\begin{array}{c} \Delta D_{local}\left(\%\right)=\frac{D\left(mc\right)-D\left(tps\right)}{D\left(tps\right)}\times 100\% \end{array}$$

(2) $$\begin{array}{c} \Delta D_{global}\left(\%\right)=\frac{D\left(mc\right)-D\left(tps\right)}{D\left(tps\right)}\times 100\% \end{array}$$

In these equations, D(mc) is the dose within the voxel determined using one MC and D (tps) is the dose from TPS with same voxel size. The prescription dose value (60 Gy) was selected to calculate the global dose difference ratio. In our analysis, voxels that overlapped with the seeds were excluded to avoid ambiguities in comparing results; these voxels are a small fraction compared to the total voxel count in the phantom.

The ROIs for skull and scalp were manually contoured on all CT slices containing the MC and TPS dose distributions. The physician‐contoured HR‐CTV, along with the skull and scalp structures, was used to calculate the dose to the tissues of interest.

Throughout this work, the doses to the tissue of interest were calculated in homogeneous settings, i.e., dose to water medium, and a heterogeneous medium, i.e., dose to non‐water medium, with identical voxel volumes, counters, and any voxels overlapping sources or outside the MC rigid were excluded.

### 2.6 Dosimetric evaluation, spatial and quantitative analysis

Dose distributions from the TPS and MC simulations were compared using DVH metrics: V_100_, V_60_, D_x_, and mean doses to structures: HR‐CTV, Skull, Skin, Right Hippocampus, Right Optic Nerve, Brainstem, and Chiasm.

Comprehensive spatial analysis was performed using isodose distributions, dose difference maps (local and global), 1D dose profiles, and 2D and 3D surface plots. The gamma analysis was performed using CERR with a defined 10% threshold, dose differences (2 %), and distance‐to‐agreement (2 mm) criteria to evaluate spatial agreement. Furthermore, the mean doses to the tissue of interest were estimated using DVH.

### 2.7 Uncertainty

Uncertainty was tracked at each stage of the MC simulation workflow. For TG‐43 characterization of the ^131^Cs source, uncertainties associated with AKS, dose‐rate constant, radial dose function, and anisotropy function were evaluated, including Type A statistical uncertainty based on the relative errors reported by the MC calculations. The film‐validation measurements included additional uncertainty contributions from film handling, source positioning, phantom material assignment, film calibration, irradiation setup [Figure S2], film processing, scanning, and dose‐response characterization.

The patient‐specific calculations included Type B uncertainties associated with CT‐to‐material assignment, HU‐to‐density conversion, CT‐number uncertainty, voxelization, phantom geometry, seed positioning, volume averaging, and MC statistical uncertainty. Because the homogeneous and heterogeneous simulations used the same benchmarked and experimentally validated source model and simulation framework, common systematic uncertainties were considered correlated and were not double‐counted when assessing the relative effect of tissue heterogeneity. The uncertainty budget included major contributions from source strength, HU‐to‐material mapping, voxelization/volume averaging, source positioning and seed reconstruction, and photon cross sections. Individual uncertainty components were estimated using AAPM TG‐138^28^ and the stoichiometric mapping approach of Schneider et al.^30^ and were combined using the root‐sum‐square method.

## 3 RESULTS

### 3.1 MC benchmarking and experimental validation

#### 3.1.1 MC benchmarking

The source strength was estimated from F4tally (MeV/gram‐particles) by appropriate unit conversion: $4.16\times 10^{-12}\;cGycm^{2}/particles$. The simulated dose rate constant was 1.059 ± 0.0013 cGyh^−1^U^−1^, which differs by only 0.3% from the published TG‐43 consensus value of (1.056 ± 0.013) cGyh^−1^U^−1^[7]. A comparison of the calculated dose rate constant from simulation with published literature values is presented in Table 7.

**TABLE 7:** Comparison of dose rate constant with published literature values for Cs‐1 Rev2 ^131^Cs source.

Author	Technique	$\Lambda \;(cGyh^{-1}U^{-1})$	Ratio with TG‐43 consensus value
Murphy et al.	TLD	0.915	0.866
Chen et al.	TLD	1.058	1.002
Whitman and Fisher	MCNP5	1.040	0.989
Rivard	MCNP5	1.046	0.991
TG‐43 Consensus	WAFAC	1.056 ± 0.013	1
This work	MCNP6.3	1.059 ± 0.0013	1.003

The radial dose function values from 0.5 cm to 7.0 cm agreed well with the published consensus data, with deviations of less than 0.7% (max. 0.77%, min. 0.11%) across the entire distance range, as shown in Figure S4 in the Supplementary Materials. The anisotropy function also demonstrated excellent agreement with the consensus values. The largest discrepancies occurred at smaller polar angles, corresponding to locations near the seed's longitudinal axis, where accurate modeling is inherently more challenging because of source geometry and self‐attenuation. Nevertheless, the average deviation across all evaluated angles (0°–90°) was only 1.2% (max. 7.1%, min. ‐4.2%), as presented in Supplementary Materials S5, confirming the accuracy of the MC source model. This proximal agreement demonstrates the accuracy of the MC source model.

We further confirmed the accuracy of our MC simulation work by comparing the dose rate at 0.25 to 7 cm from this work with available MC simulation data from Rivard et al., as presented in Table S1 in the supporting material. The average difference was 1.163% (max: 0.388%, min:‐ 1.711%).

#### 3.1.2 MC experimental validation

The MC model was validated against EBT4 Gafchromic film measurements in the heterogeneous phantom at 0.8 and 1.3 cm from the source. The measured and calculated doses were 153.24 and 142.80 cGy at 0.8 cm (6.81% difference) and 28.79 and 25.01 cGy at 1.3 cm (13.12% difference), respectively [Table 8]. The MC calculations used 2 × 10^9^ particle histories and a 0.1 cm^3^ scoring voxel. The observed agreement supported using the MC model for subsequent dose calculations.

**TABLE 8:** Comparison of absorbed dose in a heterogeneous phantom obtained from film measurement and MC simulation.

Distance [cm]	Film dose [cGy]	MC dose[cGy]	Difference (%)
0.8	153.24±4.60	142.80±3.36	6.81
1.3	28.79±0.86	25.01±0.59	13.12

### 3.2 Patient‐specific spatial and profile analysis

Figure S6–S9 in the supplementary materials compares the TG‐43‐based TPS and MC dose distributions for single—and 20‐seed configurations. For the single‐seed homogeneous water model [Figure S6], the 2D (a & b) and 3D dose distributions (c & d), horizontal and vertical profiles (e & f), and isodose contours (g) showed good agreement, with a gamma pass rate of 99.5%, an R^2^ of 1.00, and negligible differences in the dose‐difference map. Similar agreement was observed for the 20‐seed homogeneous water configuration [Figure S7], confirming consistent agreement between the TPS and MC calculations in a homogeneous material medium.

In contrast, the 20‐seed heterogeneous patient‐specific model [Figure S8] showed substantial spatial differences between TPS and MC calculations, particularly near the skull. These differences were evident in the dose maps, 3D dose surfaces, isodose contours, and horizontal and vertical profiles through the HR‐CTV, which were further supported by the dose profile drawn across the center of the HR‐CTV [Figure S9]. The dose‐difference map demonstrated localized discrepancies adjacent to the skull, while the heterogeneous TPS–MC correlation was reduced compared with the homogeneous models. These multi‐parametric analyzes demonstrate the impact of patient‐specific tissue heterogeneity on the calculated dose distribution.

### 3.3 Dose Volume Histogram (DVH) comparison

The simulation was performed in a homogeneous material configuration for benchmarking the MCNP material‐specific simulation. The DVH comparison shows close agreement between the two methods, as shown in Supplementary Material Figure S10a, for single‐ and multiple‐seed simulations [Figure 3a]. The slight discrepancies observed between MC calculations and the TPS may be attributed to variations in how each approach calculates the absorbed dose. Table 9 demonstrates close overall agreement among the DVH metrics for water calculations and disagreement for non‐water calculations. The difference in V_100_ was 0.12%, whereas V_60_ showed a slightly larger variation of 1.65%. The MC Dx values were consistently lower than those from the TPS, with differences of 4.13% for D_90_ and 5.62% for D_95_. The discrepancies observed for the OARs are likely due to regions outside the MC calculation domain.

**FIGURE 3:**
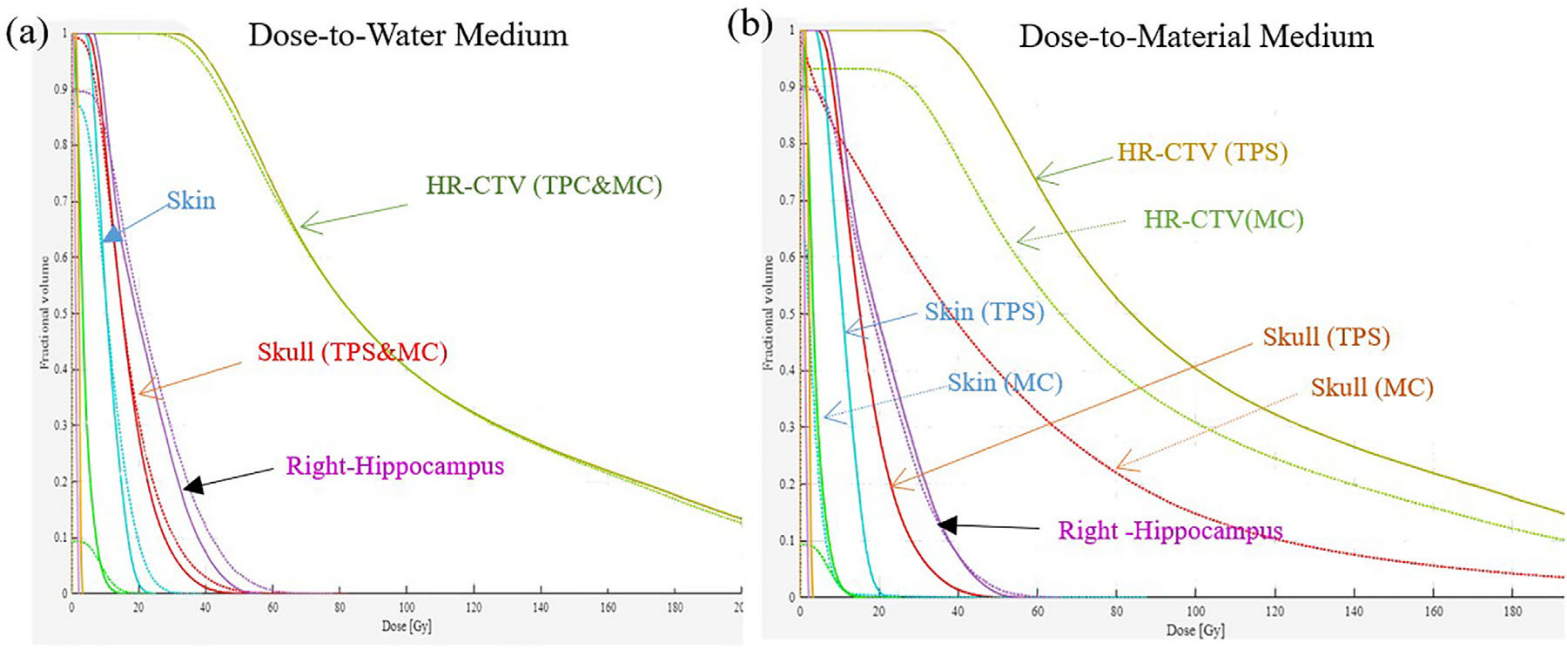
DVH comparison of TPS and MC calculations for single‐seed (a) and multiple‐seed (b) configurations under homogeneous (left panels) conditions, illustrating the benchmarking of MC dose to water medium against TPS, and heterogeneous (right panels) conditions, demonstrating the impact of tissue heterogeneity on dosimetric outcomes in material medium. Arrows indicate the DVHs of the primary structures of interest. DVHs of the remaining OARs are not labeled, as they cluster on the left side of each plot. The abscissa shows dose and the ordinate shows fractional volume.

**TABLE 9:** DVH metrics comparison.

Media	# of seeds	HR‐CTV	TPS [Gy]	MC Homogeneous [Gy]	Ratio (MC/TPS)	% diff.
Homogeneous	1	V_60_	0.01	0.01	1.00	0.00
		V_100_	0.01	0.01	1.00	0.00
		D_90_	1.15	1.17	0.98	1.74
		D_95_	1.05	1.04	1.01	0.95
	20	V_60_	0.73	0.72	1.02	1.65
		V_100_	0.41	0.40	0.99	0.12
		D_90_	45.95	44.05	1.04	4.13

		Structure	Metrics	TPS[Gy]	MC‐Heterogeneous [Gy]	Ratio (MC/TPS)	_
Heterogeneous	20	HR‐CTV	V_60_	0.73	0.55	0.76	24.42
			V_100_	0.40	0.31	0.77	23.75
			V_150_	0.46	0.35	0.76	23.91
			V_200_	0.33	0.25	0.76	24.24
			D_90_	46.01	27.85	0.61	39.47

In contrast, MC simulation in the materialized configuration revealed discernible differences in target coverage for single‐seed [Figure S10b, solid line TPS HR‐CTV, dashed line MC HR‐CTV] as well as multiple seeds, as evinced in Figure 3b, in which the olive solid line is HR‐CTV from TPS, whereas the dashed line is from MC. The solid red line is the skull DVH from MC, whereas the dashed line is from TPS, clearly showing the tissue heterogeneity effect. Similarly, the skin DVH (solid blue is from TPS and dashed is from MC) shows the heterogeneity effect. Other OAR DVHs also showed small differences, such as in the right hippocampus, as indicated by the dark arrow. The differences in OARs were nuanced due to the structure being farther from the HR‐CTV where the dose was prescribed; however, the difference observed resulted from the calculated volume not being equal. Doses, including variations in V_100_, V_60_, D_2%_, and mean doses for HR‐CTV, skull, skin, right hippocampus, right optic nerve, and brainstem, are presented in Table 9, Table 10 and Table 11. The DVH analysis showed that the TPS underestimates HR‐CTV V_60_ and V_100_ by 24% and 23%, respectively. This DVH analysis demonstrates that the presence of high‐Z skull bone significantly perturbs the isotropic dose distribution predicted by the TG‐43 formalism, necessitating model‐based dosimetry for accurate LE intracranial brachytherapy planning.

**TABLE 10:** Mean dose comparison for target and OARs.

Structure	TPS mean dose [Gy]	MC‐**homo Mean dose [Gy]	MC‐*hetero mean dose [Gy]	Ratio MC‐homo/TPS	Ratio (MC‐hetero/TPS)	MC‐homo vs TPS % difference	MC‐hetero vs TPS % difference
HR‐CTV	139.14	132.68	112.62	0.95	0.76	−4.87	−23.62
Skull	16.93	17.30	54.33	1.02	3.21	−3.04	+220.9
Skin	11.11	10.57	2.93	0.95	0.26	−4.86	−73.6
Hippocampus_R	21.84	22.49	19.87	1.03	0.91	+2.98	−9.0
Optic Nerve_R	1.33	0.05	0.05	0.04	0.04	−96.24	−96.23
Brainstem	4.04	0.94	0.79	0.23	0.20	−76.73	−80.4
Chiasm	2.32	0.05	0.05	0.21	0.02	−97.85	−97.85

*Hetero = Heterogeneous, **Homo = Homogeneous.

**TABLE 11:** Comparison of D_2%_ for selected OARs.

OARs	Volume cc	D_2%_		Heterogeneous vs Homogeneous % Difference
		MC Homogeneous [Gy]	MC Heterogeneous [Gy]	
Skull	36.63	53.85	232.85	+332.40
Skin	14.53	25.25	9.95	−60.59
Hippocampus_R	1.95	53.85	48.85	−9.29
Optic Nerve_R	17.12	0.05	0.005	−90.00
Brainstem	0.69	12.05	10.25	−14.94
Chiasm	0.40	0.05	0.01	−90.00

The significant divergence in the skull mean dose (ratio: 3.21) underscores the limitations of dose‐to‐water calculation TPS for intracranial brachytherapy. The ∼221% increase in absorbed dose within the bone in the MC model is attributed to the enhanced photoelectric cross‐section of cortical bone for ^131^Cs emissions. Furthermore, the HR‐CTV mean dose (ratio: 0.76) suggests that bone backscatter plays a non‐negligible role in target dosimetry that is currently unaccounted for in standard clinical protocols.

TPS does overestimate the dose in low‐density tissue compared with MC heterogeneous simulations. HR‐CTV demonstrates a lower mean dose in MC heterogeneous simulations, indicating realistic hotspots near seeds not captured by TPS. Hippocampus‐R shows minor variation, suggesting a moderate tissue heterogeneity impact in this region. The higher percentage difference in the mean OAR dose may be partially attributed to ROI selection. Although the TPS calculations adequately covered the OARs, the corresponding MC ROIs were not fully encompassed, leading to differences in the reported mean dose values to the selected OARs. Additional MC dosimetric investigations are warranted to further verify and strengthen the reliability of these findings.

Table 11 compares D_2%_ for both media. Incorporating tissue heterogeneity in MC significantly alters D_2%_ dose estimates compared with the homogeneous model, with the most pronounced increase observed in the skull (+ 332%), reflecting strong bone–tissue interaction effects. In contrast, soft tissues such as skin and brainstem show dose reductions (‐ 60.6% and ‐ 14.9%), indicating increased attenuation in heterogeneous media. Very small structures (optic nerve and chiasm) exhibit large reductions (‐ 90%), likely influenced by partial volume and voxel resolution effects rather than true physical dose changes. The MC Homo was estimated as the dose to the water medium, whereas MC Hetero was estimated as the dose‐to‐medium in the respective tissues, and the same structure volume was sampled in both homogeneous and heterogeneous calculations. The uncertainty is further sensitive to voxel size, contour definition, and source‐to‐bone distance.

### 3.4 Dose and isodose distributions

Figure 4 illustrates the dose and isodose distributions resulting from the TPS and MC in homogeneous and heterogeneous calculations. The close agreement between the TPS and MC homogeneous distributions, evidenced by the near‐perfect overlap of isodose lines, demonstrates the accuracy of the MC model and confirms its successful implementation for subsequent heterogeneous dose calculations.

**FIGURE 4:**
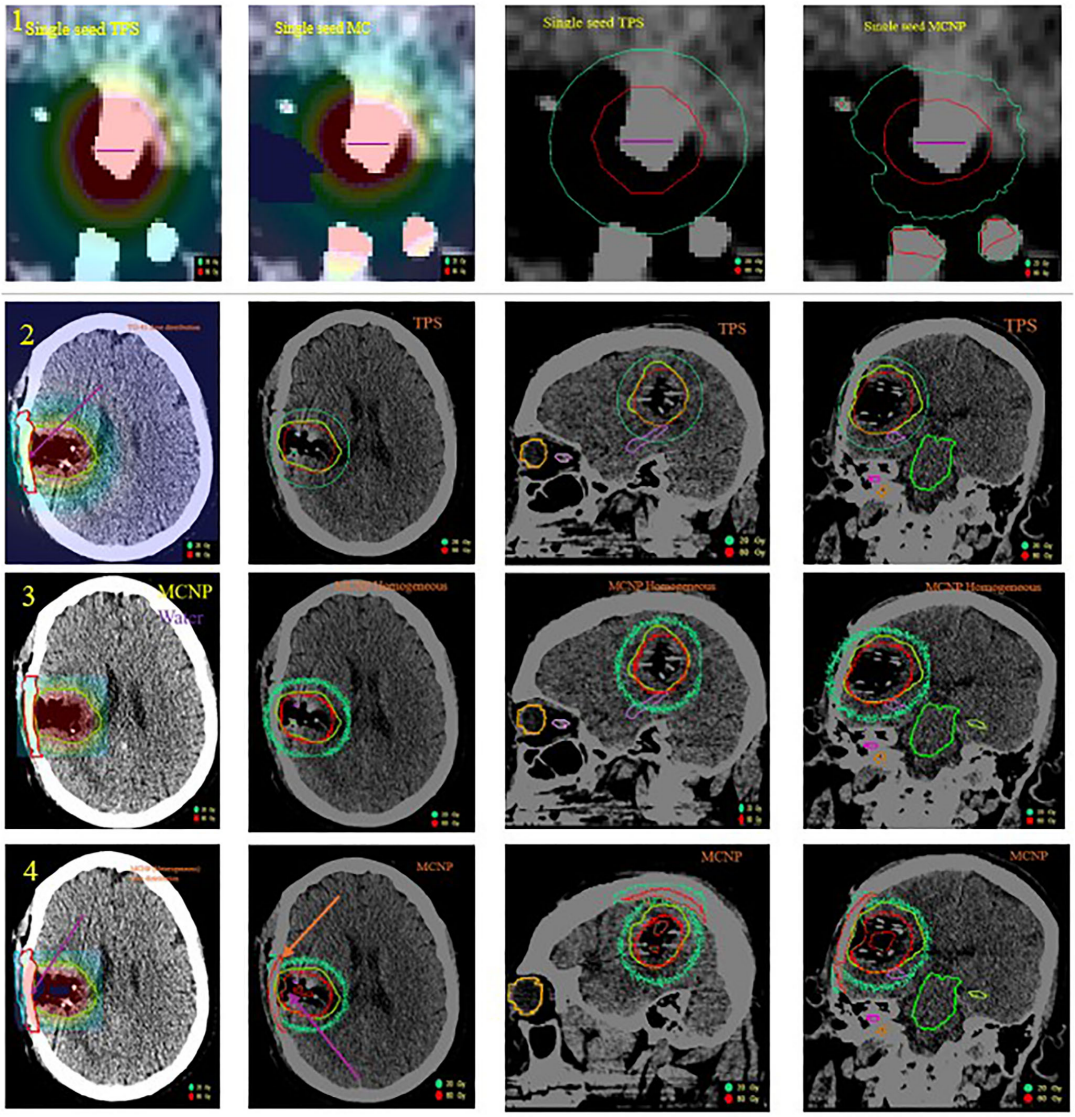
Comparison of dose and isodose distributions generated using TG‐43‐based TPS calculations and MC simulations under homogeneous and heterogeneous conditions. The 1^st^ row compares the single‐seed results from TPS and MC in heterogeneous configurations. Row 2^nd^ shows TPS‐calculated dose distributions, the 3^rd^ row presents MC results for the homogeneous model, and the 4^th^ row depicts MC dose distributions incorporating tissue heterogeneity. The yellow arrow shows the dose enhancement in the skull region, while the pink arrow shows dose shrinkage in HR‐CTV. Red and green are 60 Gy and 20 Gy isodose lines. The solid green contour is HR‐CTV.

When tissue heterogeneity was taken into account, there were dose enhancements in the high‐density skull region and bulging inward of isodose lines, as shown in the bottom row of Figure 4, which is likely attributed to photoelectric effects. The arrow indicates the heterogeneity effect.

### 3.5 Gamma analysis

Gamma analysis using a 2% dose difference and 2 mm distance‐to‐agreement criterion with a 10% dose threshold demonstrated a substantial influence of tissue heterogeneity on dose agreement as presented in Table 12. For the HR‐CTV, pass rates decreased from 100% (single seed) and 99.5% (20 seeds) under homogeneous conditions to 62.67% and 71.20%, respectively, following heterogeneity correction, indicating that material composition significantly affects dose deposition. The slightly higher pass rate for the 20‐seed configuration suggests that multiple source contributions may partially compensate for local heterogeneity‐induced perturbations. Marked reductions were also observed for the skull (99.99% to 28.58%) and skin (95.75% to 2.78%), reflecting the strong dosimetric impact of bone attenuation and tissue–air interface effects. In contrast, the right hippocampus, brainstem, optic nerve, and chiasm maintained high pass rates (> 96%) under both conditions, indicating limited sensitivity to heterogeneity within relatively uniform intracranial soft tissue. The low pass rates should be interpreted cautiously, as volume‐averaging, partial‐volume sampling, and steep dose gradients may have disproportionately influenced gamma outcomes, contributing to apparent disagreement beyond true dosimetric differences.

**TABLE 12:** Gamma pass rates (%) obtained using a 2%/2 mm gamma criterion with a 10% dose threshold for the HR‐CTV and selected organs at risk under homogeneous and heterogeneous dose calculation conditions. Results are presented for single‐seed and 20‐seed HR‐CTV configurations. The heterogeneous calculations demonstrate the influence of tissue composition on dose agreement, particularly in structures adjacent to bone and tissue–Air interfaces.

Structure	Number of Seeds	Gamma pass rate (10% threshold)	
		2%/2 mm	
		Homogeneous	Heterogeneous
HR‐CTV	1	100.00%	62.67%
	20	99.5%	71.20%
Skull		99.99%	28.58%
Skin		95.75%	2.78%
Hippocampus_R		96.99%	97.13%
Optic Nerve_R		99.12%	98.12%
Brainstem		98.20%	97.98%
Chiasm		99.35%	99.21%

### 3.6 Global and local percentage dose differences

Local dose difference maps highlighted significant deviations between TPS and MC near high‐density bone regions, while global dose differences indicated an overall bias in TPS calculations.

The global difference $(\Delta D_{global}$), [Figure 5, left] exhibited a high degree of convergence (0.06% ± 2.90%), validating the MC source model against the clinical TPS baseline. However, the local difference ($\Delta D_{local}$) [Figure 5, right] demonstrates an extremely wide variance (18.57% ± 382.66%), which quantifies the failure of the water‐equivalent TG‐43 formalism to resolve tissue‐specific interactions. The significant local discrepancies were attributed to the photoelectric effect within the cortical bone, leading to localized dose enhancement and distal shielding that were not captured by standard clinical planning algorithms. Figures 5b, Figure 5c, and 5d display the corresponding histograms of dose difference for HR‐CTV, Skin, and Skull, respectively.

**FIGURE 5:**
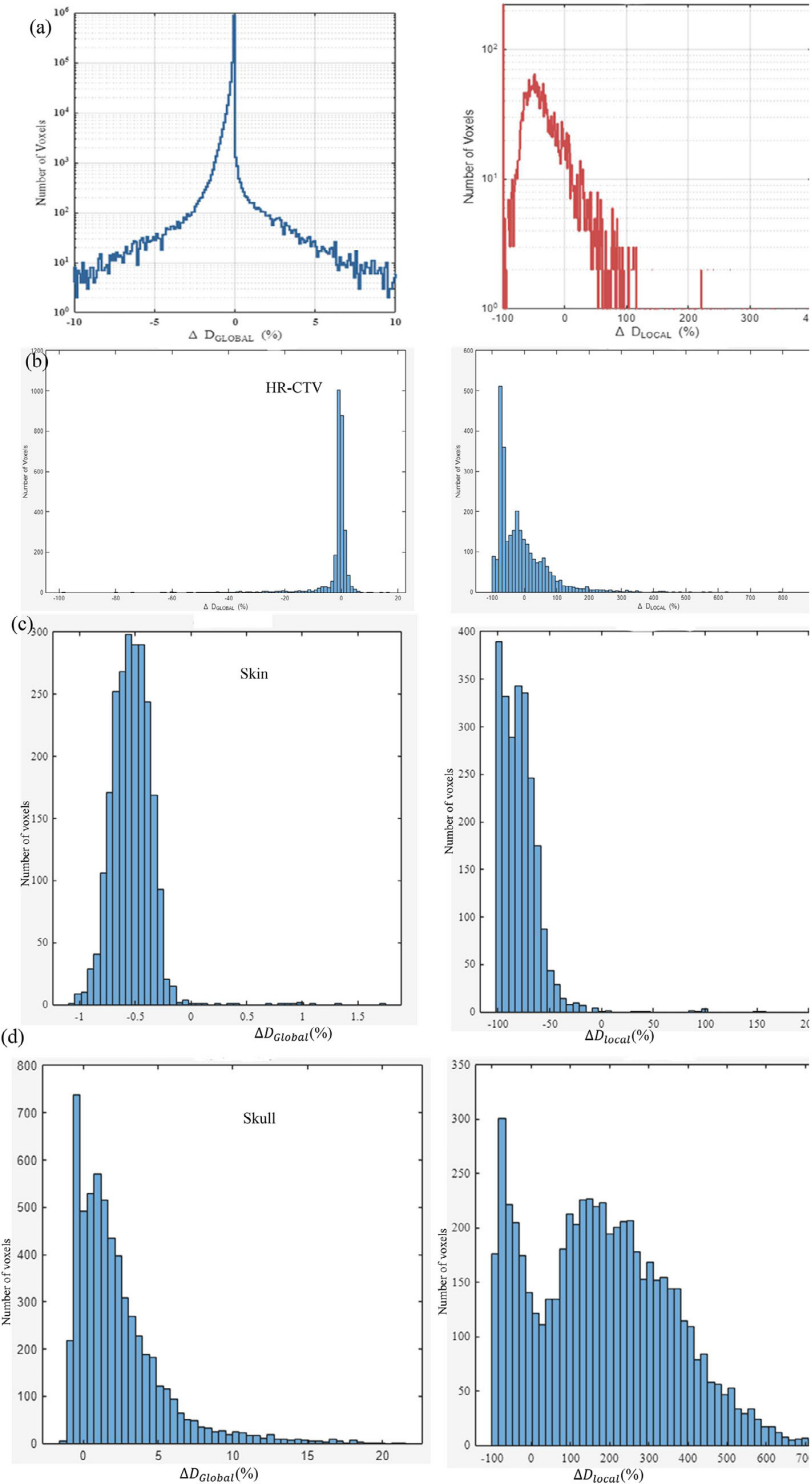
Frequency distribution of global versus local dose differences. The narrow global difference distribution (right) confirms robust baseline agreement and source calibration. Conversely, the wide, skewed local difference distribution (left) quantifies the failure of water‐equivalent TPS algorithms to resolve localized dose enhancement (> 300%) and attenuation effects inherent to the high‐Z intracranial environment. Abscissa is (ΔD%) and the ordinates are the number of voxels. a. Local vs global MC‐TPS baseline validation, b. HRCTV, c. Skin and d. Skull frequency distribution of global versus local dose differences.

The dose difference analysis demonstrated that tissue heterogeneity has a pronounced impact on dose deposition in regions adjacent to the skull and bone–tissue interfaces. While the HR‐CTV exhibited modest global dose differences (2.01%), substantial local variations (10.05% ± 315.71%) were observed due to skull‐induced photon backscatter. Similarly, the skull showed significant local dose enhancement (124.03% ± 132.87%), primarily driven by increased photoelectric absorption in calcium‐rich bone. These results highlight the importance of accounting for heterogeneity effects in intracranial ^131^Cs brachytherapy dosimetry.

### 3.7 Uncertainty analysis

All simulations for patient‐specific dosimetry were performed using 1 × 10^9^ particle histories to maintain low statistical uncertainty in the calculated dose distributions, including high‐gradient regions. In the HR‐CTV, the mean MC statistical uncertainty was < 0.5%. At the bone–brain interface, where a 3.21‐fold dose enhancement was observed, the relative statistical uncertainty remained < 1.5%. At distal OARs, attenuation and shielding reduced photon fluence and resulted in higher relative uncertainties of approximately 3%–5%. The statistical uncertainty also varied with voxel size, contour definition, and source‐to‐tissue distance.

The components contributing to the total uncertainty included source strength (± 2%),^31^ HU‐to‐material mapping (± 3.5%),^30^ voxelization/volume averaging (± 2%),^8^ source positioning/seed reconstruction (± 1.5%)[8], and photon cross sections (± 1.5%).^29^ Assuming these components were independent, their combined standard uncertainty was approximately 5.0% based on the root‐sum‐square method. The MC statistical uncertainty was evaluated separately and remained below the reported limits for the clinically relevant dose regions. These results indicate that the observed heterogeneity‐related dose differences were larger than the MC statistical uncertainty in the primary regions of interest.

## 4 DISCUSSION

This study evaluated the dosimetric impact of tissue heterogeneity in intracranial ^131^Cs brachytherapy by comparing conventional TG‐43–based TPS calculations with patient‐specific MC simulations. The results demonstrated reasonable agreement between TPS and MC calculations under homogeneous water‐equivalent conditions, thereby validating the source modeling, simulation framework, and computational methodology. However, clinically meaningful discrepancies emerged once realistic tissue heterogeneity was introduced into the simulations.

The dose‐to‐water medium calculation results confirmed that the implemented MC model accurately reproduced TG‐43 benchmark dosimetry. The close agreement in DVH metrics, dose profiles, isodose distributions, and gamma‐index analyzes demonstrated that the MC model reliably reproduced the expected reference dose distributions. These findings establish that the observed discrepancies in heterogeneous simulations arise primarily from tissue‐composition effects rather than computational or geometrical inconsistencies within the MC framework.

In contrast, the heterogeneous patient‐specific simulations revealed substantial deviations between the TPS and MC calculations. The largest discrepancies were observed near high‐density structures, particularly the skull, where photoelectric interactions significantly altered local dose deposition. Because ^131^Cs emits relatively low‐energy photons, photon transport becomes highly sensitive to variations in material composition and effective atomic numbers. Consequently, the TG‐43 assumption of an infinite homogeneous water medium becomes increasingly inaccurate in anatomically complex intracranial environments; however, this depends upon the distances from the implanted sources due to the inverse square effect.

The DVH analysis demonstrated reductions in target coverage metrics, including V_100_, V_60_, and D_95_, when heterogeneity corrections were incorporated. These findings suggest that TG‐43–based treatment planning may overestimate HR‐CTV coverage in clinical practice. The substantial reduction observed in D_95_ is particularly significant because this metric is commonly used to evaluate the adequacy of target coverage. Therefore, the results indicate that the actual dose delivered to portions of the HR‐CTV may be lower than predicted by conventional TPS calculations.

The slight discrepancy observed in the HR‐CTV DVH shown in Figure 3 may be attributable to differences in seed orientation between the calculations. However, the V_60_ and V_100_ values are in close agreement for both methods, indicating that this discrepancy has a negligible impact on the overall accuracy of the MC calculations. This conclusion is further supported by the dose distribution comparisons presented in Figures 4 and S6 & S7 in supporting material, which demonstrate close agreement between the MC and TPS results.

At the same time, the MC simulations demonstrated substantial dose enhancement within the skull region, with mean skull doses increasing by more than 220% relative to TG‐43 estimates, as observed in Figure 4 (4^th^ row) and Figure S9 in supporting material. This enhancement is primarily attributable to increased photoelectric absorption and backscatter within cortical bone. The high atomic number and density of bone significantly increase the probability of photon interactions for low‐energy ^131^Cs emissions, producing localized increases in absorbed dose near bone–tissue interfaces; however, dose overestimation occurs in some patient cases when the cavity is close to the skull. Note that the MC homogeneous calculation was estimated as dose‐to‐water medium, whereas MC heterogeneous was estimated as dose‐to‐medium in respective tissues, and the same structure volume was sampled in both homogeneous and heterogeneous calculations.

Dose distribution differences observed in Figure 4 for the single‐seed simulation are attributable to the assignment of HU values for air. The threshold was defined such that all voxels within the skull region were classified as brain soft tissue. Consequently, the air‐filled resection cavity was not explicitly represented in the material assignment, leading to an apparent cutoff in the dose distribution within these regions. This limitation does not significantly affect the accuracy of the calculations because the primary purpose of the single‐seed simulation in the materialized phantom was to demonstrate the presence of heterogeneity effects. In other words, the simulation served as a validation of the heterogeneous phantom model rather than as a quantitative assessment of dose within the resection cavity.

In the DVH analysis of the HR‐CTV for the heterogeneous configuration [Figure 3b], the MC‐generated DVH may appear not to be normalized to unity. However, upon closer inspection, the curve does in fact originate at a normalized volume of one. The unusual shape of the HR‐CTV DVH may be related to the dose bulging into the surrounding brain tissue, as observed in Figure 4, where the isodose lines from the MC calculation curve further into the brain tissue compared with those generated by the TPS. The underlying cause of this behavior remains unclear and warrants further investigation. Figures S10a,&b in supporting material, and Figure 3a benchmark the accuracy of the model, while Figure 3b presents the discernible difference in the effect of tissue heterogeneity, especially for HR‐CTV, Skull, and Skin.

The larger discrepancies observed for the OARs, as presented in Tables 10 & 11, can be attributed primarily to the MC dose calculation regions and the relatively large source‐to‐structure distances, which resulted in higher statistical uncertainties. The uncertainty is further sensitive to voxel size, contour definition, and source‐to‐tissue distance. In particular, the MC ROI may have only partially encompassed certain OARs, including the right hippocampus, right optic nerve, brainstem, and optic chiasm, leading to lower dose estimates compared with those calculated by the TPS. For example, the MC‐calculated dose to the brainstem in the homogeneous phantom was 0.94 Gy, compared with 0.79 Gy in the heterogeneous phantom. Similarly, the dose to the right hippocampus was 22.49 Gy in the homogeneous phantom and 19.87 Gy in the heterogeneous phantom. In contrast, the dose to the optic chiasm remained unchanged at 0.05 Gy in both phantom models. Furthermore, the gamma analysis results [Table 12] indicate that the observed dose differences for these OARs were not clinically significant. Although the TPS generally overestimated the dose relative to the MCNP calculations, these discrepancies are small and are unlikely to have any meaningful impact on treatment evaluation or clinical outcomes.

The impact of tissue heterogeneity on OAR dosimetry varied considerably among structures, with the minimal changes observed in the right hippocampus (96.99% vs 97.13%), while the brainstem exhibited a smaller change of 0.22%; in contrast, no heterogeneity‐induced effect was observed for the right optic nerve or chiasm, whose dose differences remained unchanged between homogeneous and heterogeneous models, as presented in Table 12, which is likely due to the selection of the calculation region of interest in both TPS and MC.

These findings may have clinical implications for dose assessment near cranial bone structures. Enhanced backscatter and altered photon transport near the skull may increase local dose deposition in adjacent tissues; however, the clinical significance of these effects will depend on the source‐to‐skull geometry, tissue composition, and dose distribution. The observed differences indicate that conventional TG‐43‐based calculations may not fully characterize dose deposition in regions adjacent to the skull, highlighting the potential value of heterogeneity‐aware dose calculations for patient‐specific dosimetric assessment.

The spatial analyzes further emphasized the limitations of homogeneous dose‐calculation assumptions. The isodose contour distortions, local dose‐difference maps, and one‐dimensional dose profiles consistently demonstrated that the effects of heterogeneity are highly localized and therefore not fully captured by global dosimetric metrics alone. In particular, the local dose‐difference analysis revealed substantial voxel‐level deviations despite relatively small global dose differences, highlighting the importance of spatially resolved dosimetric evaluation in intracranial brachytherapy.

Gamma‐index analysis provided additional evidence of heterogeneity‐induced discrepancies. While homogeneous phantom simulations produced near‐perfect gamma pass rates, the heterogeneous phantom simulations showed substantial reductions in agreement near high‐gradient and high‐density regions, which is also supported by the global and local dose differences [Figure 5]. These results reinforce the conclusion that tissue heterogeneity significantly perturbs dose deposition patterns and cannot be neglected in patient‐specific intracranial dosimetry.

The present study also demonstrates the value of combining multiple complementary evaluation approaches, including DVH analysis, gamma‐index assessment, dose‐difference mapping, surface visualization, and profile‐based comparisons. Although DVHs remain clinically important, they do not fully characterize localized spatial dose perturbations. The additional spatial analyzes performed here provided important insight into the underlying physical mechanisms responsible for the observed dosimetric discrepancies.

Limitations of this study should also be acknowledged. First, although the phantom geometry was derived from the patient's post‐implant CT data, the voxelized phantom was generated by assigning each anatomical structure (brain tissue, skull, and scalp) the corresponding ICRU‐44 reference elemental composition and mass densities [Table 5] rather than using voxel‐by‐voxel CT‐based material and density assignment. This approach was intentionally adopted to minimize uncertainties associated with CT number‐to‐density calibration, image noise, partial volume effects, and artifacts from the implanted ^131^Cs seeds, while preserving the dominant anatomical heterogeneity relevant to low‐energy photon transport. For ^131^Cs photons, tissue composition and the pronounced differences between cortical bone and soft tissue are expected to have a greater influence on dose deposition than the relatively small density variations within the given tissue type. Therefore, the reported dosimetric differences are expected to be governed primarily by tissue composition and major anatomical interfaces, particularly the skull‐brain interfaces, rather than intra‐tissue density variations, such as those associated with cerebrospinal fluid spaces, edema or variations in bone density, which may introduce small local dose perturbations. Future investigations incorporating patient‐specific voxel‐by‐voxel material and density assignments may further improve dosimetric accuracy and enable a more comprehensive assessment of tissue heterogeneity effects.

Second, the present work focused primarily on post‐implant dosimetric evaluation rather than prospective treatment optimization. Integration of MBDCAs directly into clinical treatment‐planning workflows represents an important future direction. In addition, an important limitation of the present study is that the patient‐specific analysis was derived from a single post‐implant clinical dataset. Accordingly, the quantitative differences reported here should be interpreted as a proof‐of‐concept demonstration of heterogeneity effects in one clinically realistic geometry and should not be assumed to represent the magnitude of these effects across the broader patient population. The magnitude and spatial distribution of these differences are expected to depend on patient anatomy, implant geometry, source‐to‐skull distance, and tissue composition. Studies involving larger patient cohorts are therefore required to establish the range, reproducibility, and clinical significance of these effects.

The third limitation is the assignment of only four material classes (bone, brain tissue, skin, and air) to phantom voxels from single post‐implant patient data. Although the dominant heterogeneities affecting low‐energy ^131^Cs photon transport were represented, voxel‐by‐voxel CT‐based material mapping could improve local dose accuracy, particularly near tissue interfaces and high‐gradient regions. Further study is warranted utilizing more patient data and different MC codes.

Fourth, statistical uncertainty in the MC calculations was controlled by using 1 × 10^9^ particle histories. Note that we simulated 2 × 10^9^ histories for MC benchmarking and validation, and 1 × 10^9^ for patient‐specific dosimetry. This is why the statistical uncertainties appear higher in the latter case. Residual uncertainty was primarily associated with source and geometric modeling, CT‐based tissue‐material assignment, source positioning, and experimental dosimetry. Because the homogeneous and heterogeneous calculations used the same benchmarked and experimentally validated source model and simulation framework, common systematic uncertainties were expected to largely cancel when assessing the relative effect of tissue heterogeneity. Therefore, the observed differences in DVH metrics, spatial dose distributions, dose profiles, and gamma‐analysis results are primarily interpreted as effects of tissue heterogeneity rather than independent uncertainties between the two dose calculations. Nevertheless, additional studies using larger numbers of histories and multiple patient datasets are warranted to further reduce statistical uncertainty and assess the generalizability of these findings.

Despite these limitations, the results demonstrated that tissue heterogeneity has a substantial impact on dose distributions in intracranial ^131^Cs brachytherapy. The findings further indicate that MBDCA, particularly MC methods, may provide clinically meaningful improvements in dosimetric accuracy relative to conventional TG‐43–based planning approaches.

Clinically, these deviations may influence both target coverage and the dose to critical structures such as the scalp, hippocampus, and brainstem. Although TG‐43 remains widely used due to its simplicity and computational efficiency, the findings of this study highlight the potential benefits of incorporating heterogeneity‐aware dose calculation methods, MBDCA, in clinical practice. The increased calculated dose in the skull may be relevant to assessment of radiation effects in adjacent cranial tissues; however, the present dosimetric study does not provide clinical outcome data and therefore cannot establish an association with osteoradionecrosis, radionecrosis, or other toxicity. Evaluation of such clinical consequences will require larger patient cohorts together with outcome‐based analyzes. Nevertheless, careful consideration is required when reporting and utilizing the current TG‐43 protocol for ^131^Cs implant into the intracranial lesion.

## 5 CONCLUSION

This study developed, validated, and applied a patient‐specific MC framework to quantify the dosimetric impact of tissue heterogeneity in intracranial ^131^Cs Tile‐based intracranial brachytherapy. In homogeneous settings, the HR‐CTV V_100_ and V_60_ differences were only 0.12% and 1.65%, whereas in heterogeneous settings, the corresponding V_60_ and V_100_ differences increased to 24.42±4.97%, and 23.75±4.97%, respectively. Similarly, the mean dose differences for the skull and scalp were 3.04% and 4.86 % in a homogeneous water medium, but increased dramatically to 220.90±4.97% and 23.62±4.97%, respectively, when patient‐specific tissue heterogeneity was incorporated. Complementary dosimetric analyzes, including gamma evaluation, isodose distributions, dose‐difference maps, and spatial dose profiles, consistently demonstrated that the TG‐43 formalism overestimated target coverage by approximately 23.00±4.97% while underestimating dose to the skull by approximately 221.00±4.97%. Collectively, these results demonstrate that neglecting patient‐specific tissue heterogeneity can lead to clinically meaningful inaccuracies in dose estimation for low‐energy intracranial brachytherapy.

Beyond quantifying these dosimetric effects, this work provides a validated patient‐specific MC framework and a set of reference test cases that can facilitate the commissioning and verification of model‐based dose calculation algorithms for intracranial brachytherapy. The findings support the adoption of MC—or MBDCA‐based dose calculations to improve the accuracy of treatment evaluation and post‐implant dosimetry in permanent low‐energy ^131^Cs Tile‐based permanent intracranial brachytherapy. Future studies should investigate how these dosimetric differences influence clinically relevant outcome metrics, including target control, normal tissue toxicity, and treatment response.

## AUTHOR CONTRIBUTIONS

All authors contributed to the study conception and design. Tirthraj Adhikari designed the study and developed the manuscript; Jae W. Jung designed the study and reviewed the manuscript; Farhad Jafari, Sherif Gadoue, Puja Sharma, and Tomas Montenegro reviewed the manuscript; and Clara Ferreira designed the study and reviewed the manuscript. Tirthraj Adhikari carried out the Monte Carlo simulation data for the work. The first draft of the manuscript was written by Tirthraj Adhikari. Final approval of the work to be published was finished by Tirthraj Adhikari.

## CONFLICT OF INTEREST STATEMENT

The authors declare no conflicts of interest.

## ETHICAL STATEMENT

This study was conducted in accordance with the ethical standards of the institutional research committee and the principles of the Declaration of Helsinki. The study involved retrospective analysis of de‐identified post‐implant CT/DICOM data. Institutional review board approval was obtained for the use of this data. During the writing of this manuscript, available AI‐based language tools were used to improve readability. All scientific content, analysis, and conclusions are the original work of this study.

## Supporting information




**Supporting Information**: acm270872‐sup‐0001‐SupMat.docx

## Data Availability

The de‐identified data are available from the corresponding author upon reasonable request and with appropriate institutional approval, subject to applicable ethical and privacy requirements.
