# Supplementary material for: Impact of tissue heterogeneity on intracranial Cesium‐131 brachytherapy dosimetry: A patient‐specific Monte Carlo Study
Source: J Appl Clin Med Phys. 2026 Oct 29;27(11):e70872. doi: 10.1002/acm2.70872 (PMC13720234; doi:10.1002/acm2.70872)
Supplement: Supplementary file 1 [file ACM2-27-e70872-s001.docx]

**Supporting Materials**

This document provides a broader range of methods, materials, and graphical analyses to supplement the findings in the original study.

Figure S1. MC experimental geometrical setup for the simulation of AKS, dose rate constant, radial, and anisotropy constants. Note that AKS was estimated in dry air.

**a**

b

^131^Cs source

1^st^ 5 mm bone phantom

2^nd^ 5 mm bone phantom

5 mm SW phantom over 2^nd^ bone phantom

0.3 mm SW on the 5 cm phantom that mimics collagen

5 cm Solid water phantom

to account for backscattering

**c**

Figure S2. a. Film measurement experimental setup for benchmarking the MC; b. ^131^Cs source held at the center of SW; and c. MC experimental geometry identical to the film setup for tally scoring. The voxel size in the film and the tally-scoring voxel in MCNP were identical.

Resection Cavity

seed

seed

Figure S3. Material universe assignment verification. Left: original CT slice; right: material-specific brain model.

Radial dose function

| Distance (cm) | Radial Dose Function(RDF) | TG-43 Consensus RDF |
| --- | --- | --- |
| 0.5 | 1.004 | 1.006 |
| 1 | 1 | 1 |
| 1.5 | 0.964 | 0.962 |
| 2 | 0.909 | 0.908 |
| 3 | 0.78 | 0.777 |
| 4 | 0.644 | 0.642 |
| 5 | 0.522 | 0.518 |
| 6 | 0.412 | 0.411 |
| 7 | 0.324 | 0.323 |

Figure S4. Comparison of radial dose function as a function of distances with TG-43 consensus values.

Anisotropy function at r= 0.5 cm

AF[-]

| Polar angle (degree) | Tg-43 Consensus | This work |
| --- | --- | --- |
| 0 | 0.829 | 0.860073 |
| 10 | 0.678 | 0.701935 |
| 20 | 0.778 | 0.794654 |
| 30 | 0.868 | 0.877413 |
| 40 | 0.923 | 0.928258 |
| 50 | 0.958 | 0.961155 |
| 60 | 0.978 | 0.97979 |
| 70 | 0.99 | 0.991013 |
| 80 | 0.998 | 0.997816 |
| 90 | 1 | 1 |

Anisotropy functions at r=1 cm

AF[-]

| Polar anngle (degree) | TG-43 consensus | This work |
| --- | --- | --- |
| 0 | 0.829 | 0.905471 |
| 10 | 0.678 | 0.73194 |
| 20 | 0.778 | 0.818756 |
| 30 | 0.868 | 0.888322 |
| 40 | 0.923 | 0.932257 |
| 50 | 0.958 | 0.961641 |
| 60 | 0.978 | 0.980895 |
| 70 | 0.99 | 0.992102 |
| 80 | 0.998 | 0.997418 |
| 90 | 1 | 1 |

Anisotropy function at r= 2 cm

AF[-]

| Polar angle (degree) | TG-43 consensus | This work |
| --- | --- | --- |
| 0 | 0.838 | 0.889658 |
| 10 | 0.752 | 0.77289 |
| 20 | 0.832 | 0.845832 |
| 30 | 0.894 | 0.903318 |
| 40 | 0.935 | 0.940588 |
| 50 | 0.963 | 0.965533 |
| 60 | 0.981 | 0.982875 |
| 70 | 0.993 | 0.993558 |
| 80 | 0.999 | 0.999139 |
| 90 | 1 | 1 |

Anisotropy function at r= 3 cm

AF[-]

| Polar angle (degree) | TG-43 consensus | This work |
| --- | --- | --- |
| 0 | 0.838 | 0.875761 |
| 10 | 0.776 | 0.796371 |
| 20 | 0.847 | 0.859235 |
| 30 | 0.902 | 0.909316 |
| 40 | 0.939 | 0.943881 |
| 50 | 0.964 | 0.967986 |
| 60 | 0.982 | 0.982963 |
| 70 | 0.993 | 0.993835 |
| 80 | 0.999 | 0.998635 |
| 90 | 1 | 1 |

Anisotropy function at r= 5 cm

AF[-]

| Polar angle (degree) | TG-43 consensus | This work |
| --- | --- | --- |
| 0 | 0.843 | 0.879061 |
| 10 | 0.801 | 0.819046 |
| 20 | 0.862 | 0.872189 |
| 30 | 0.91 | 0.915543 |
| 40 | 0.943 | 0.948249 |
| 50 | 0.966 | 0.968859 |
| 60 | 0.982 | 0.983537 |
| 70 | 0.993 | 0.993957 |
| 80 | 0.999 | 0.99823 |
| 90 | 1 | 1 |

Anisotropy function at r= 7 cm

AF[-]

| Polar angle (degree) | TG-43 consensus | This work |
| --- | --- | --- |
| 0 | 0.853 | 0.885395 |
| 10 | 0.815 | 0.82955 |
| 20 | 0.868 | 0.87859 |
| 30 | 0.912 | 0.919051 |
| 40 | 0.944 | 0.94865 |
| 50 | 0.967 | 0.968853 |
| 60 | 0.982 | 0.983561 |
| 70 | 0.993 | 0.993629 |
| 80 | 0.998 | 0.999891 |
| 90 | 1 | 1 |

Figure S5. Anisotropy function of this work with TG-43 consensus values.

d

c

Figure S6. Spatial dose distribution and corresponding profile comparison for the single-seed simulation, demonstrating close agreement between the present simulation results and the TPS baseline.

Figure S7. Multi-parametric validation of the MC transport model against the TG-43 based clinical TPS. (a–b) 2D dose distributions at the central transverse slice (z = 67); (c–d) 3D dose surface topography illustrating the high-gradient resolution of the MC tally; (e–f) 1D horizontal and vertical dose profiles passing through the maximum dose centroid, demonstrating peak alignment; (g) Isodose contour overlay for 30, 60, and 120 Gy (from in to out) levels showing volumetric conformity; (h) Pixel-by-pixel dose difference map (TPS - MCNP) highlighting the impact of inter-source attenuation within the tile; (i) Dose correlation scatter plot confirming high linearity and the absence of systematic spatial or rotational bias between calculation algorithm.

(a) TPS dose Map

Figure S8. Multi-parametric comparison of the MC calculation taking into account the material heterogeneity against TG-43-based TPS calculation. The tissue heterogeneity effect is evidenced by dose map (a-TPS, b-MC),3d surface dose(c-TPS, d-MC), profiles (e &f), Isodose comparison (g), dose difference, and correlation (i).

Figure S9. Profile comparison across the center of HR-CTV.

Figure S10. DVH comparison of TPS and MCNP calculations for single-seed (a, b) and multiple-seed (c, d) configurations under homogeneous (left panels) conditions, illustrating the benchmarking of MC dose to water medium against TPS, and heterogeneous (right panels) conditions, demonstrating the impact of tissue heterogeneity on dosimetric outcomes in material medium. Arrows indicate the DVHs of the primary structures of interest. DVHs of the remaining OARs are not labeled, as they cluster on the left side of each plot. The abscissa shows dose, and the ordinate shows fractional volume.

Table S1. MC simulation output comparison with available MC data (dose-to-water).

| Distance [cm] | MC Output (This work) | | Rivard et al. | % diff |
| --- | --- | --- | --- | --- |
| [cm] | [MeV/g-hist] | Relative Error | [MeV/g/-hist |  |
| 0.25 | 3.75E-03 | 2.00E-04 | 3.71E-03 | -1.28% |
| 0.30 | 2.73E-03 | 2.00E-04 | 2.70E-03 | -1.05% |
| 0.50 | 1.07E-03 | 4.00E-04 | 1.06E-03 | -1.13% |
| 0.75 | 4.87E-04 | 6.00E-04 | 4.81E-04 | -1.33% |
| 0.80 | 4.29E-04 | 6.00E-04 | 4.25E-04 | -0.89% |
| 1.00 | 2.75E-04 | 8.00E-04 | *2.72E-04* | -0.98% |
| 1.30 | 1.60E-04 | 1.00E-03 | 1.59E-04 | -1.06% |
| 1.50 | 1.18E-04 | 1.20E-03 | 1.17E-04 | -0.89% |
| 2.00 | 6.29E-05 | 2.10E-03 | 6.24E-05 | -0.86% |
| 2.50 | 3.76E-05 | 2.10E-03 | 3.72E-05 | -1.09% |
| 3.00 | 2.41E-05 | 2.60E-03 | 2.38E-05 | -1.26% |
| 3.50 | 1.62E-05 | 3.20E-03 | 1.59E-05 | -1.71% |
| 4.00 | 1.12E-05 | 3.80E-03 | 1.10E-05 | -1.66% |
| 4.50 | 7.99E-06 | 4.50E-03 | 7.86E-06 | -1.58% |
| 5.00 | 5.80E-06 | 5.30E-03 | 5.713E-06 | -1.53% |
| 5.50 | 4.27E-06 | 6.20E-03 | 4.22E-06 | -1.14% |
| 6.00 | 3.19E-06 | 7.20E-03 | 3.15E-06 | -1.42% |
| 6.50 | 2.39E-06 | 8.30E-03 | 2.38E-06 | -0.39% |
| 7.00 | 1.83E-06 | 9.50E-03 | 1.82E-06 | -0.84% |
